# Heeding the voices of nurses: a systematic review and meta-analysis of organizational silence levels among clinical nurses

**DOI:** 10.1186/s12912-025-03138-1

**Published:** 2025-05-16

**Authors:** Shasha Wen, Wenting Ji, Di Gao, Xianying Lu, Ting Zhao, Jing Gao, Ran Xu

**Affiliations:** 1 School of Nursing, Chengdu University of Traditional Chinese Medicine, No. 1166, Liutai Avenue, Wenjiang District, Chengdu, Sichuan Province 611137 China; 2 Affiliated Hospital of Chengdu University of Traditional Chinese Medicine, Chengdu, Sichuan Province China

**Keywords:** Nurses, Organizational silence, Systematic review, Meta-analysis

## Abstract

**Background:**

Systematic integration of nursing staff perspectives constitutes a critical determinant of organizational efficacy. However, more and more studies showed that organizational silence occurred among nurses frequently. We aimed to analyze the pooled level of organizational silence in nursing field so as to provide the evidence for the nursing management.

**Methods:**

The data were collected following predefined inclusion criteria. Two researchers independently screened studies, extracted data and assessed the quality of included studies. Stata 15.1 was used to examine the data.

**Results:**

There were 34 studies in total, involving 13,394 nurses from China, Turkey, and Korea. Nurses had a moderate level of organizational silence in our study, with an average mean score of 2.69 (95% *CI*: 2.57–2.81). Significant heterogeneity in the prevalence estimates of the included studies was detected (*I*^*2*^ = 99.00%, *P* < 0.001). Regression analysis did not reveal the source of heterogeneity. Subgroup analysis revealed that only the oncology department was a source of heterogeneity.

**Conclusions:**

Our findings revealed a moderate level of organizational silence among nursing professionals, offering critical insights into their unmet communicative needs within healthcare institutions. This empirical evidence underscores the necessity to develop targeted intervention strategies aimed at counteracting the detrimental impacts of organizational silence on both clinical safety outcomes and long-term institutional sustainability.

**Supplementary Information:**

The online version contains supplementary material available at 10.1186/s12912-025-03138-1.

## Introduction

Nurses as the frontline caregivers of patients, plays a vital role in meeting the growing demand for healthcare services in organizations and enabling high-quality nursing services to run smoothly [1]. However, study [2] demonstrated that the global nursing workforce, currently estimated at fewer than 28 million professionals worldwide, is projected to face a deficit of 4.6 million by 2030, a gap maybe exacerbated by the persistent systemic barriers in organizational management [3], with increasing numbers of nurses reporting that they may experience difficulty in voicing their concerns [4]. The presence of organizational silence was strongly associated with increased nurse turnover [5, 6].

The concept of organizational silence was initially developed within the domain of organizational behavior studies [7]. It delineates the collective phenomenon in which employees refrain from articulating their opinions on organizational matters, opting instead for more circumspect methods of communication [8]. With the progression of society and additional research on organizational silence, interdisciplinary integration between nursing science and management science has led scholars to apply this phenomenon to discussions on healthcare management. In the field of nursing, organizational silence refers to the fact that nurses could have put forward their own ideas, suggestions and views to their nursing organizations based on their experience and knowledge, so as to improve and enhance some aspects of the work of their nursing organizations, but they choose to retain their views, or refine and filter their views for various reasons [8]. Excessive organizational silence can have a negative impact on organizations and individuals. Specifically, the organization’s silence blocks the flow of information and often leads managers to receive delayed and false information, thereby affecting their ability to make timely and effective decisions [9, 10]. Besides, it also limits progress and reform within the care team [11]. The motivation of the nurse decreases over time when the nurses’ views are ignored, or when the organization fails to acknowledge or provide feedback in a group environment. This would cause nurses to thereby have a feeling of helplessness, meaninglessness, hopelessness, and other negative emotions [12], further reducing the nurses’ sense of team belonging and professional identity [13], lowering job satisfaction [14], and leading to various forms of job burnout [15, 16]. In severe cases, this can even lead to nurses considering leaving the profession [17], increasing the risk of brain drain and ultimately jeopardising the stability of the nursing team [18]. Although, there is some evidences demonstrated that an appropriate level of silence can have a favorable impact, for example, lessen the probability of internal conflict, maintaining the stability an d order of the organization, and simplifying the decision-making process [19], *m*uch research had determined that the deleterious effects of organizational silence exceed the potential advantages [20, 21]. A systematic evaluation revealed that merely two of the 192 studies delineated the benefits of organizational silence [22]. It is evident that understanding the overall degree of organizational silence among the current nursing workforce is critical to mitigating the adverse effects.

In recent years, there has been a growing body of research investigating organizational silence among nurses. While the phenomenon has gained increasing academic attention, substantial discrepancies persist in reported levels of organizational silence across studies. For instance, Zhang Shaoguo et al. [23] inquired 358 nurses and found that the mean score of organizational silence was (3.41 ± 0.58), which is at a high level. In contrast, Meng Bing et al. [24] evaluated 323 pediatric nurses and found that the mean score of organizational silence of nurses was (1.97 ± 0.76) which was at a low level. Adding to the inconsistency, Eun-Young doool [25] examined 301 hospital nurses discovered the mean score of nurses’ organizational silence to be (2.42 ± 0.53), which is at a moderate level. These conflicting findings highlight the current lack of consensus in the field. Notably, despite the growing number of primary studies, there remains a critical gap in quantitative systematic evaluations to reconcile these disparities. This evidence gap underscores the necessity for comprehensive synthesis. Therefore, this study aims to consolidate existing evidence through systematic analysis, thereby providing a more precise quantification of organizational silence levels among nursing professionals.

## Methods

The systematic review and meta-analysis were performed according to Preferred Reporting Items For Systematic Reviews and Meta-analysis (PRISMA) guidelines [26], and the checklist could be found in Table 1. The study has a PROSPERO registration number (CRD42023474612). Since this study was a meta-analytic review, ethics committee permission was not required.

**Table 1:** The characteristics of 34 studies included in this meta-analysis

Study (first author, publication year)	Country	Department	Sample size	Male	Female	Age mean (M ± SD)	Scale	Mean scores^1^		Mean scores^2^	
								Mean^1^	SD^1^	Mean^2^	SD^2^
Yin Z D (2020)	China	oncology	404	37	367	NA	NOSB	49.25	16.11	2.46	0.81
Yang S N (2017)	China	ICU	205	48	157	NA	ESB	2.83	0.51	2.83	0.51
Meng B X (2023)	China	pediatric	323	0	323	NA	NOSB	39.49	15.23	1.97	0.76
Sun H S (2022)	China	Multi department	200	21	179	NA	NOSB	56.71	10.97	2.84	0.55
Chang J Y (2018)	China	Mixed	287	12	275	24.25 ± 1.34	ESB	3.20	0.49	3.20	0.49
Qi J F (2023)	China	operating	395	143	252	32.68 ± 6.17	NOSB	49.31	15.83	2.47	0.79
Zhang W X (2017)	China	Multi department	473	17	456	NA	NOSB	47.16	14.55	2.36	0.73
Liu H (2019)	China	Multi department	406	11	395	NA	ESB	2.27	1.25	2.27	1.25
Cheng H H (2019)	China	Multi department	1012	9	1003	30.16 ± 6.99	ESB	34.50	9.91	2.88	0.83
Jin L M (2017)	China	Multi department	485	26	459	NA	NOSB	47.50	15.04	2.38	0.75
Lu J H (2021)	China	Multi department	546	27	519	NA	NOSB	53.75	15.65	2.69	0.78
Ma J W (2020)	China	Multi department	437	46	391	NA	ESB	2.67	0.68	2.67	0.68
Yang J (2016)	China	Multi department	807	30	777	NA	NOSB	61.89	12.53	3.09	0.63
Wang Q H (2016)	China	Multi department	262	NA	NA	NA	ESB	29.25	6.62	2.44	0.55
Zhang A M (2018)	China	Multi department	485	26	459	NA	NOSB	47.50	15.04	2.38	0.75
Zhang S G (2017)	China	Multi department	358	21	337	NA	NOSB	68.31	11.55	3.41	0.58
Li Y (2023)	China	Multi department	458	12	446	NA	NOSB	54.88	13.88	2.74	0.69
Wang P C (2023)	China	emergency	210	33	177	NA	NOSB	54.70	15.02	2.74	0.75
Wang F H (2016)	China	psychiatry	603	26	577	NA	ESB	37.66	6.46	3.14	0.54
Chen L (2022)	China	gynecology and obstetrics	207	NA	NA	32.34 ± 10.34	NOSB	44.88	16.45	2.24	0.82
Huang M H (2020)	China	Multi department	464	0	464	NA	ESB	33.84	8.57	2.82	0.71
Wang D H (2018)	China	Multi department	451	5	446	NA	ESB	33.78	8.66	2.82	0.72
Zhong T (2016)	China	Multi department	603	26	577	30.22 ± 6.88	ESB	3.13	0.54	3.13	0.54
Cao R (2019)	China	neurology	262	14	248	NA	ESB	33.25	10.80	2.77	0.90
Meng W (2020)	China	Multi department	226	11	215	27.29 ± 5.87	NOSB	48.69	15.38	2.43	0.77
Zhou A Q (2022)	China	operating	235	41	194	NA	NOSB	47.34	17.14	2.37	0.86
Wang Y (2017)	China	operating	405	46	359	NA	NOSB	52.04	14.97	2.60	0.75
Yuan C J (2023)	China	pediatric	201	5	196	NA	ESB	36.21	8.75	3.02	0.73
Xiong J (2019)	China	oncology	278	NA	NA	NA	NOSB	51.27	17.28	2.56	0.86
Eun-Young Dool (2019)	Korea	Multi department	301	8	293	26.75 ± 3.69	OSS	2.42	0.53	2.42	0.53
Serap Parlar Kılı (2020)	Turkey	Multi department	671	132	539	29.39 ± 7.20	OSS	3.18	0.79	3.18	0.79
G. Kaya (2021)	Turkey	Multi department	341	67	274	NA	OSS	2.85	0.59	2.85	0.59
Fedayi Ya˘gara (2023)	Turkey	Multi department	224	23	201	NA	OSS	2.90	0.61	2.90	0.61
Aslan SK (2020)	Turkey	Multi department	169	32	137	NA	OSBS	2.77	0.92	2.77	0.92

Abbreviations: NA = not avaliable. Scale: ESB = Employee silence behavior designed by Zheng Xiaotao (2008), NOSB = Nurses’ organizational silence behavior designed by YangJing (2016), OSS = Organizational Silence Scale developed by Van Dyne, Ang(2003), OSBS = Organizational Silence Behavior Scale designed by Yalçın(2016). 1: The score before the conversion 2: The score after the conversion

### Search strategy

Two independent researchers (WSS and JWT) searched the following database: including PubMed, Cochrane, Embase, CINAHL, Web of Science, Sinomed, China National Knowledge Infrastructure (CNKI), China Scientific and Technical Periodicals Index (VIP), and Wanfang Data electronic databases (from launch to September 2023). The search terms comprised including (“nurses” OR “nurse” OR “personnel” OR “nursing” OR “nursing personnel” OR “registered nurses” OR “registered nurse”) AND (“organizational silence” OR “organizational silent”) were used without date restrictions. Furthermore, a manual screening was conducted on all references of the included studies to identify any further eligible research that could not be obtained. Appendix 1 displays the search approach that was employed.

### Selection criteria

All abstracts and full-text were independently reviewed by two researchers (WSS and JWT). Any disagreement will be dissolved by discussion until reaching consensus or by consulting GJ.

To be included in the meta-analysis, the studies should (1) the participants were registered nurse; (2) the study design was observational, including cross-sectional, case-control, and cohort studies; and (3) reporting data on the status of nurses’ organizational silence (M ± SD). Exclusion criteria were (1) studies of which complete data were not available, or of which data could not be analyzed; (2) articles with duplicated data, with only the most recent articles selected; (3) studies in languages other than English or Chinese; and (4) studies for which full text was not available.

### Data extraction

Two authors (WSS and JWT) did the literature search and quality assessment independently. The literature screening process was as follows: the first step was to exclude duplicate studies; the second step was to read the titles and abstracts to exclude clearly irrelevant articles based on the inclusion criteria; and the third step was to read the full text to further determine its applicability.

Two reviewers (WSS and JWT) developed and used a structured questionnaire to extract the study data independently, and disagreements, if any, were resolved through discussion. Data extraction included: authors, year of publication, country, assessment scale with its mean and standard deviation, sample characteristics (age (m ± sd), gender, sample size, department).

### Quality assessment

The final included articles were appraised for their methodological quality using the observational studies using Joanna Briggs Institute (JBI) Checklist for Analytical Cross Sectional Studies [27]. Two reviewers (WSS and JWT) independently assessed the quality of the included articles. Articles were scored into four categories, “yes,” “no,” “unclear,” and “not applicable,” in terms of the following aspects “: (1) clearly defined criteria for inclusion in the sample; (2) described the study population and setting in detail; (3) measured exposure in a valid and reliable manner; (4) used objective and standardized criteria to measure the condition; (5) identified confounders; (6) described strategies to deal with the confounders; (7) measured the outcome in a valid and reliable manner; and (8) used appropriate statistical analysis. If the answer is “yes,” the item is scored 1 point; otherwise, it is scored 0 points. The quality score is the total number of “yes” answers and ranges from 0 to 8, with higher scores indicating higher quality. Any disagreements in the data were resolved by a third party GJ.

### Data synthesis and statistical analysis

Because the included studies used more than one version of the assessment scale, the number of questions and total scores varied. Therefore, to make the data from the meta-analysis comparable and analyzable, we entered the mean scores and standard deviations of the scales into the meta-analysis as corrected mean scores and standard deviations (uniformly referred to as mean scores and standard deviations in this review). For studies that provided only total mean scores, the data were converted as follows: total mean score and standard deviation were divided by the number of items in the scale, respectively. Two reviewers (WSS and JWT) transformed the data individually and then cross-checked it, and any discrepancies were resolved by consultation with the GJ.

The meta-analysis was conducted using stata15.1. We pooled the mean scores and standard deviations of six scale scores across nurses, and pooled mean scores were presented with weighted effect sizes and 95% confidence intervals (*CI*). Heterogeneity was assessed by the *I*^*2*^ statistic and *P*-value, with *I*^*2*^ values of 25%, 50%, and 75% indicating low, moderate, and high heterogeneity, respectively [28]. When *I*^*2*^ > 50% and *p* < 0.10, it indicated that there was moderate or high heterogeneity, and the random effect model was used for analysis; otherwise, the fixed effect model was used. When heterogeneity occurred, meta regression and subgroup analysis were performed to assess the source of heterogeneity. Meta-regression analysis was performed to assess the potential effect of important covariates that may lead to heterogeneity. In this study, meta-regression and subgroup analysis were performed to explore whether the differences on the type of age, sample size, years, geographic location, and assessment scale, when covariates were statistically significant, with *P* ≤ 0.05. Meanwhile, sensitivity analysis, funnel plots, Begg’s test and Egger’s test were used to detect publication bias.

### Informed consent

This systematic review aims to synthesize and evaluate the evidence regarding the levels of organizational silence among clinical nurses. As this study does not involve the collection of new data or interventions with individuals, ethical committee approval and informed consent are not required.

## Results

### Study selection

**Fig. 1:**
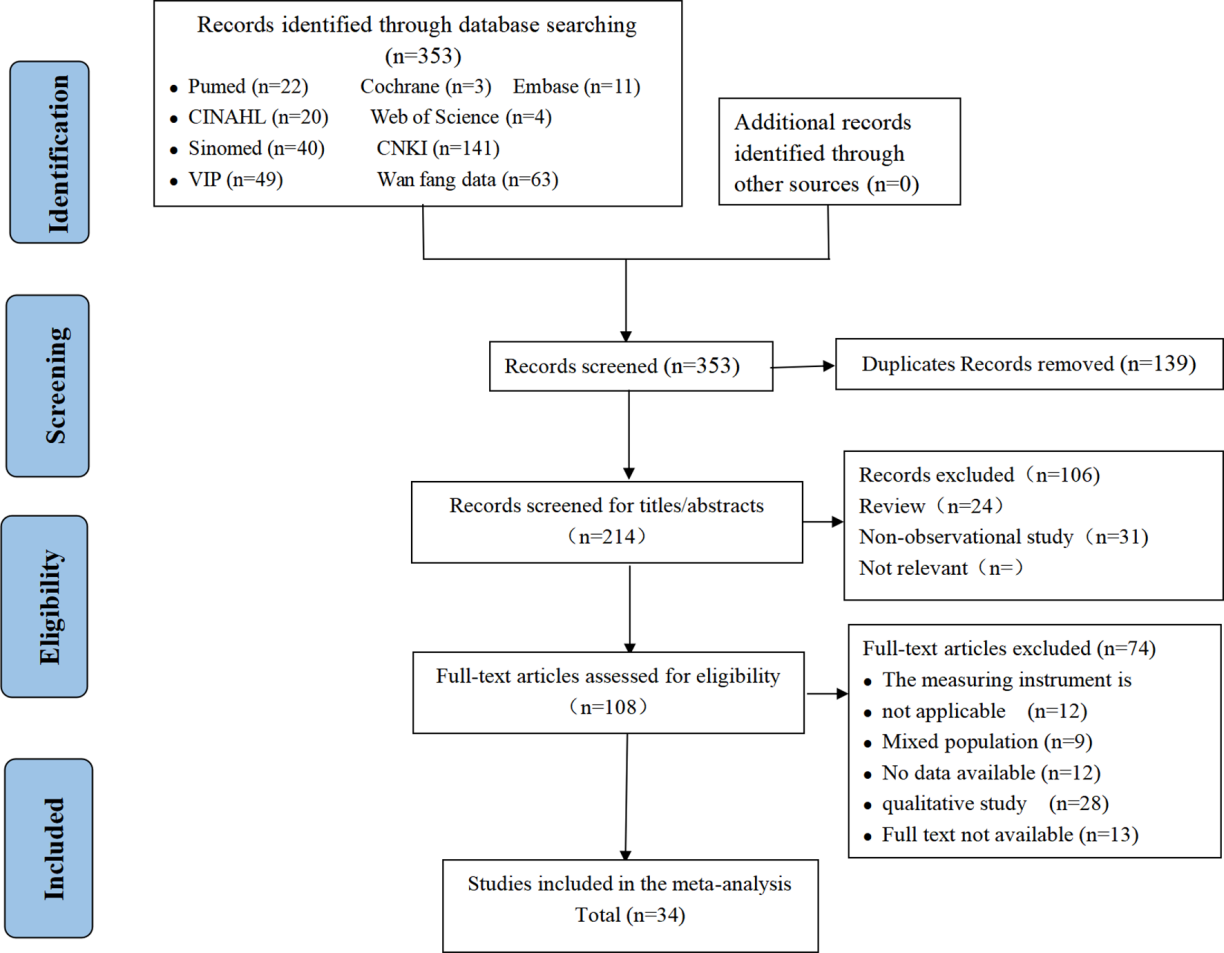
PRISMA flow chart for the study screening process

The search identified 353 relevant studies from 9 databases; 139 studies were excluded because of duplication, and 214 studies were excluded because of low relevance. Finally, 108 studies were selected for full-text screening. After reviewing the full texts, 34 articles met the eligibility criteria. The reasons for exclusion and the process of exclusion are detailed in Fig. 1. Table 1 lists the basic characteristics of the included studies.

### Study characteristics

A total of 34 studies were conducted across 29 studies in China, four in Turkey, and one in Korea, including 13,394 nurses (ranging from 169 to 1012), of which 11,692 were female, 955 were male and 747 nurses did not report gender. All the studies were cross-sectional studies that adopted validated self-report questionnaires as tools for measuring outcomes. Various tools for measuring nurses’ organizational silence have been utilized in individual studies: Nurses’ organizational silence behavior by Yang Jing 2016 (NOSB), employee silence behavior by Zheng Xiao 2008 (ESB), the organizational silence behavior scale by yalçın 2016 (OSBS), and the organizational silence scale developed by Van Dyne, Ang 2003 (OSS). The mean scale of all measurements ranged from 1 to 5 and was consistently used in all studies to measure the level of organizational silence in nurses. The data were transformed since 25 studies reported only total mean scores. When the quality of studies was measured by the JBI, all studies were judged as having a low risk of bias.

### Quality assessment results

The quality assessment found all studies to be at low risk of bias (score ranged from 6 to 8), indicating that the included studies were of acceptable quality. The risk of bias in included studies stemmed mainly form item 6 (inappropriate strategies for dealing with confounders), item 7 (unclear ways of measuring outcomes), and item 8 (inappropriate use of statistical analysis). The evaluation of the risk of bias of the included studies is presented in Table 2.

**Table 2:** Quality assessment of 34 studies included in this systematic evaluation

Studies	1. Were criteria for inclusion in the sample clearly defined?	2. Were the study subjects and the setting described in detail?	3. Was the exposure measured in a valid and reliable way?	4. Were objective, standard criteria used for measurement of the condition?	5. Were confounding factors identified?	6. Were strategies to deal with confounding factors stated?	7. Were the outcomes measured in a valid and reliable way?	8. Was appropriate statistical analysis used?	Score
Yin Z D (2020)	Yes	Yes	Yes	Yes	Yes	Yes	Yes	Yes	8
Yang S N (2017)	Yes	Yes	Yes	Yes	Yes	Yes	Yes	Yes	8
Meng B X (2023)	Yes	Yes	Yes	Yes	Yes	Yes	Yes	Yes	8
Sun H S (2022)	Yes	Yes	Yes	Yes	Yes	Yes	Yes	Yes	8
Chang J Y (2018)	Yes	Yes	Yes	Yes	Yes	Unclear	Yes	Unclear	6
Qi J F (2023)	Yes	Yes	Yes	Yes	Yes	Yes	Yes	Yes	8
Zhang W X (2017)	Yes	Yes	Yes	Yes	Yes	Yes	Yes	Yes	8
Liu H (2019)	Yes	Yes	Yes	Yes	Yes	Unclear	Unclear	Yes	6
Cheng H H (2019)	Yes	Yes	Yes	Yes	Yes	Unclear	Yes	Unclear	6
Jin L M (2017)	Unclear	Yes	Yes	Yes	Yes	Unclear	Yes	Yes	6
Lu J H (2021)	Yes	Yes	Yes	Yes	Yes	Yes	Yes	Yes	8
Ma J W (2020)	Yes	Yes	Yes	Yes	Yes	Yes	Yes	Yes	8
Yang J (2016)	Yes	Yes	Yes	Yes	Yes	Unclear	Yes	Yes	7
Wang Q H (2016)	Yes	Yes	Yes	Yes	Yes	Yes	Unclear	Yes	7
Zhang A M (2018)	Yes	Yes	Yes	Yes	Yes	Yes	Yes	Yes	8
Zhang S G (2017)	Unclear	Yes	Yes	Yes	Yes	Unclear	Yes	Yes	6
Li Y (2023)	Yes	Yes	Yes	Yes	Yes	Yes	Yes	Yes	8
Wang P C (2023)	Yes	Yes	Yes	Yes	Yes	Yes	Yes	Yes	8
Wang F H (2016)	Yes	Yes	Yes	Yes	Yes	Yes	Unclear	Yes	7
Chen L (2022)	Yes	Yes	Yes	Yes	Yes	Unclear	Yes	Unclear	6
Huang M H (2020)	Unclear	Yes	Yes	Yes	Yes	Yes	Unclear	Yes	6
Wang D H (2018)	Unclear	Yes	Yes	Yes	Yes	Yes	Yes	Yes	7
Zhong T (2016)	Unclear	Yes	Yes	Yes	Yes	Yes	Unclear	Unclear	5
Cao R (2019)	Yes	Yes	Yes	Yes	Yes	Yes	Unclear	Yes	7
Meng W (2020)	Yes	Yes	Yes	Yes	Yes	Yes	Yes	Yes	8
Zhou A Q (2022)	Yes	Yes	Yes	Yes	Yes	Yes	Yes	Yes	8
Wang Y (2017)	Yes	Yes	Yes	Yes	Yes	Yes	Yes	Yes	8
Yuan C J (2023)	Yes	Yes	Yes	Yes	Yes	Yes	Yes	Unclear	7
Xiong J (2019)	Yes	Yes	Yes	Yes	Yes	Yes	Yes	Yes	8
Eun-Young Dool (2019)	Yes	Yes	Yes	Yes	Yes	Yes	Unclear	Yes	7
Serap Parlar Kılı(2020)	Yes	Yes	Yes	Yes	Yes	Yes	Yes	Yes	8
G. Kaya (2021)	Yes	Yes	Yes	Yes	Yes	Yes	Unclear	Yes	7
Fedayi Ya˘gara (2023)	Unclear	Yes	Yes	Yes	Yes	Yes	Unclear	Yes	6
Aslan SK (2020)	Unclear	Yes	Yes	Yes	Yes	Yes	Yes	Yes	7

### Meta-analysis

#### Pooled status of nurses’ organizational silence

**Fig. 2:**
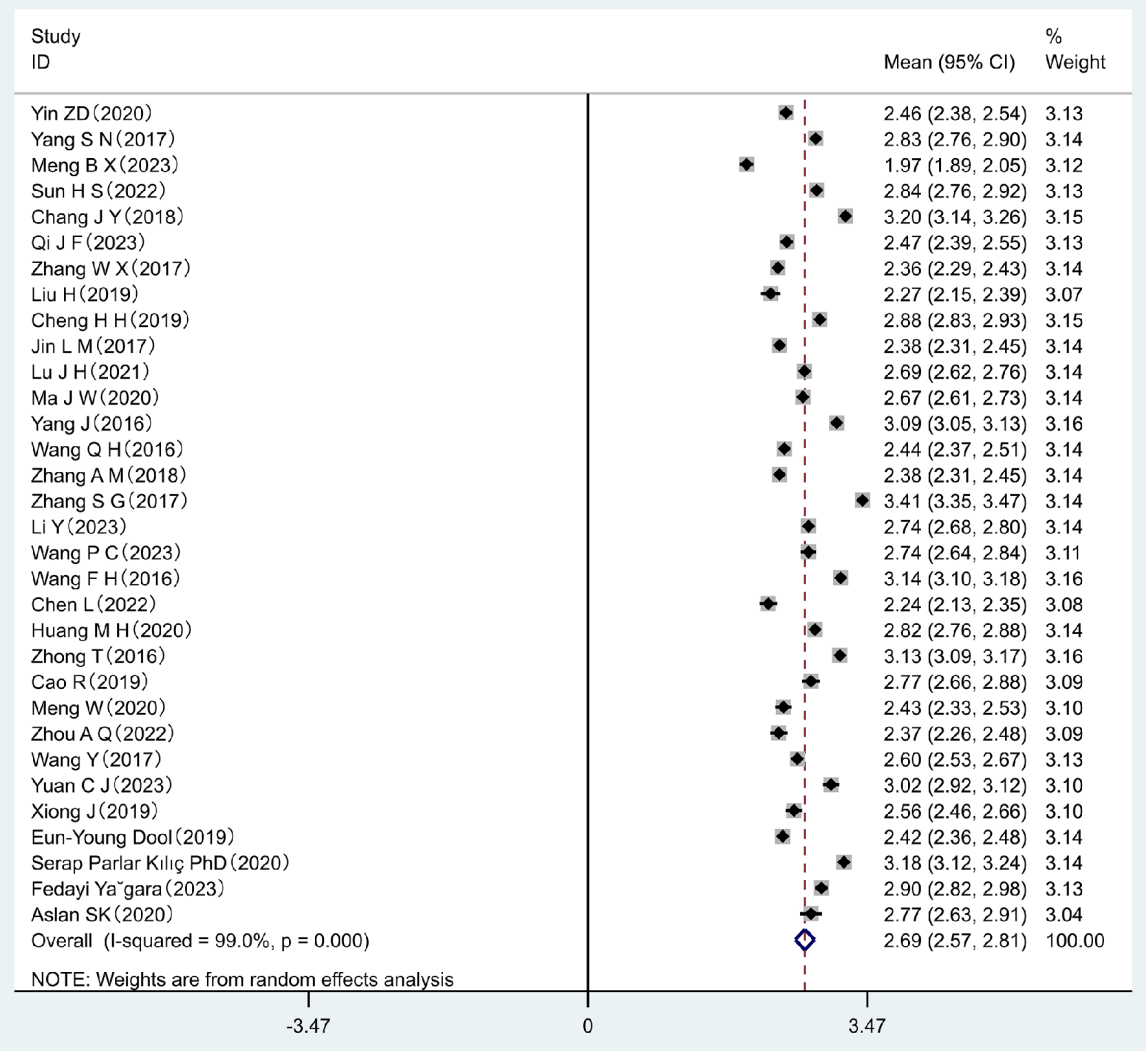
Forest plot of pooled mean scores for levels of organizational silence among clinical nurses

A total of 34 studies were included in the meta-analysis. The results of the heterogeneity test revealed high heterogeneity in the data (*I*^2^ = 99.0%, *p* < 0.001). Using a random-effects model, the pooled mean score of nurses’ organizational silence was 2.69 (95% *CI*: 2.57–2.81). Based on a Likert rating scale of 5 out of 5, nurses’ organizational silence was at a moderate level. The pooled status of the nurses’ organizational forest plot is shown in Fig. 2.

#### Meta-regression and subgroup analysis of nurses’ organizational silence

A high degree of heterogeneity was found after meta-analysis of the results (*I*^*2*^ = 99.0%, *P* < 0.001). We performed meta-regression, subgroup analysis and sensitivity analysis to determine the cause of heterogeneity. Sample size (*P* = 0.988), year of publication (*P* = 0.995), country (*P* = 0.997), assessment scale (*P* = 0.997), and department (*P* = 1.000) were selected as covariates. Meta-regression analysis revealed that all the variables were greater than 0.05, indicating that sample size, year of publication, country, assessment scale, and department were not sources of heterogeneity (see Table 3). As shown in Table 4, subgroup analysis revealed that sample size, country, and department were statistically significantly different between groups (all with *P*^*a*^<0.05) and were associated with the level of organizational silence among nurses. However, scale (*P*^*a*^=0.06) and year of publication (*P*^*a*^=0.30) were not significantly different between groups and were statistically significant within groups (all with *P*^*b*^ <0.05).

**Table 3:** Meta-regression of nurses’ organizational silence

The variables	*SE*	*Z*	95% *CI*	*P*
Sample size	0.665	-0.02	-1.313 ~ 1.293	0.988
Publication year	0.608	0.01	-1.187 ~ 1.196	0.995
Country	1.408	0.00	-2.764 ~ 2.754	0.997
Assessment scale	0.903	0.00	-1.770 ~ 1.770	0.997
Department	0.465	0.02	-0.911 ~ 0.911	1.000

**Table 4:** Subgroup analysis of nurses’ organizational silence

Subgroup	Number of studies	Sample size	Pooled mean score (95% CI)	Model	*P* ^a^	Heterogeneity		
						*I*^*2*^ (%)	*Q*	*P* ^b^
Sample size					0.01*			
<300	13	2988	2.70(2.55,2.85)	Random		97.8	553.45	< 0.001
300–500	15	6186	2.58(2.41,2.74)	Random		98.9	1304.06	< 0.001
>500	6	4271	2.95(2.77,3.14)	Random		98.9	518.29	< 0.001
Publication year					0.30			
<2019	12	5453	2.82(2.60,3.03)	Random		99.4	1672.66	< 0.001
2019–2021	13	5517	2.68(2.54,2.81)	Random		97.8	525.88	< 0.001
2022–2023	9	2475	2.59(2.38,2.79)	Random		98.1	476.37	< 0.001
Country					< 0.01*			
China	29	11,739	2.68(2.56,2.80)	Random		98.9	2816.81	< 0.001
Turkey	4	1405	2.93(2.75,3.11)	Random		95.2	72.22	< 0.001
South Korea	1	301	2.42(2.36,2.48)	Random		-	-	-
Scale					0.06			
ESB	12	5222	2.83(2.68,2.99)	Random		96.7	694.99	< 0.001
NOSB	17	6517	2.57(2.42,2.72)	Random		96.8	1654.37	< 0.001
OSS	4	1537	2.84(2.53,3.15)	Random		96.9	315.18	< 0.001
OSBS	1	169	2.77(2.63,2.91)	Random		-	-	-
Department					< 0.01*			
multi department	22	5849	2.76(2.64,2.89)	Random		98.9	1965.95	< 0.001
pediatric	2	524	2.26(1.69,2.84)	Random		98.7	78.25	< 0.001
operating	3	1035	2.70(2.37,3.02)	Random		97.8	73.86	< 0.001
tumor	2	682	2.43(2.39,2.48)	Fixed		0	0.63	0.430
others	5	1538	2.68(2.37,2.99)	Random		98.8	357.49	< 0.001

Note: *P*^*a*^ value for the between-subgroup difference; *P*^*b*^ value for the heterogeneity within subgroups by *Q* test; *P < 0.05

#### Sensitivity analysis

**Fig. 3:**
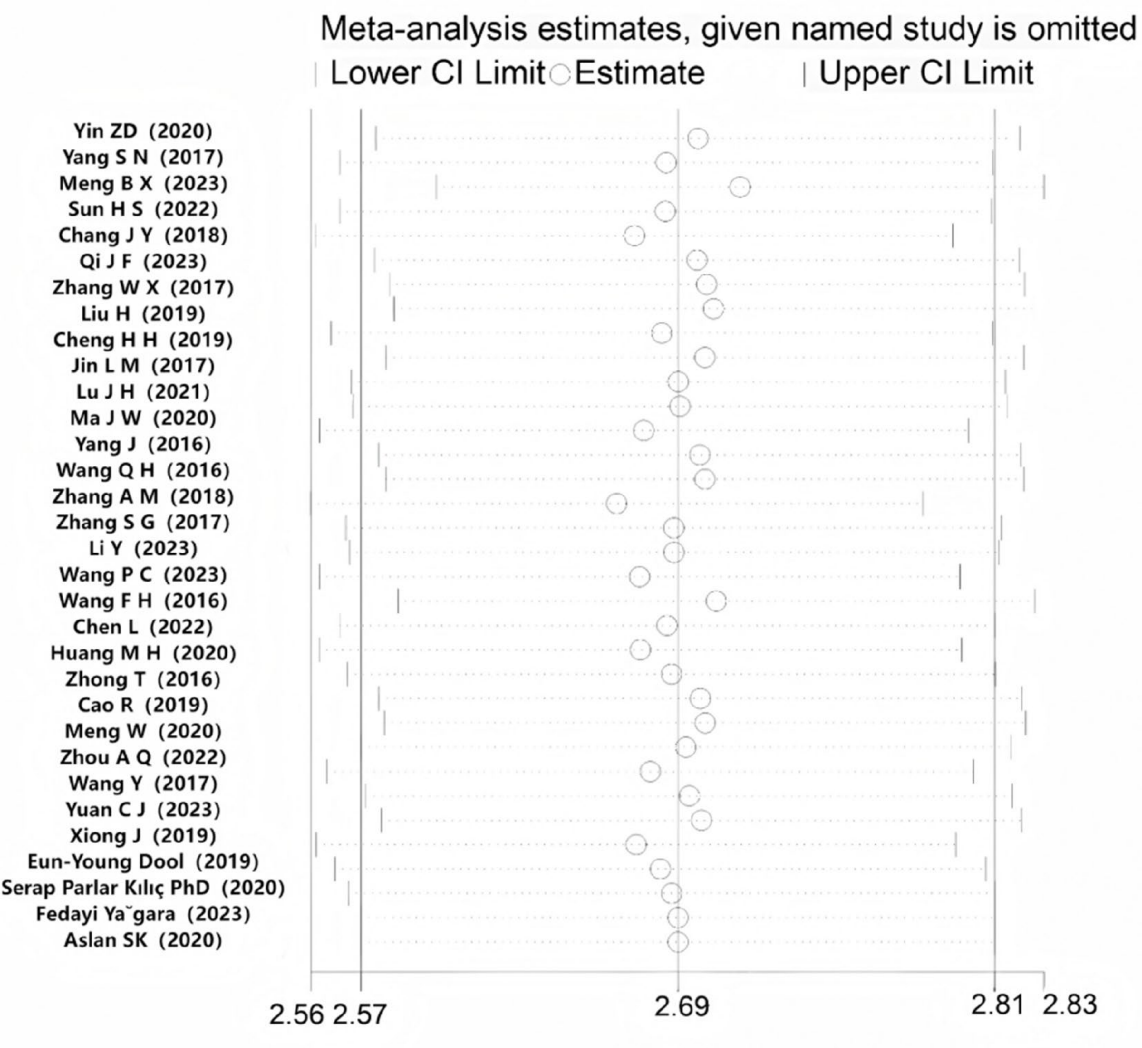
Sensitivity analysis of the total score of nurses’ organizational silence

The sensitivity analysis results were not significantly different from the overall composite estimates, indicating that the meta-analysis results were relatively stable and reliable. (See Fig. 3).

#### Publication bias

**Fig. 4:**
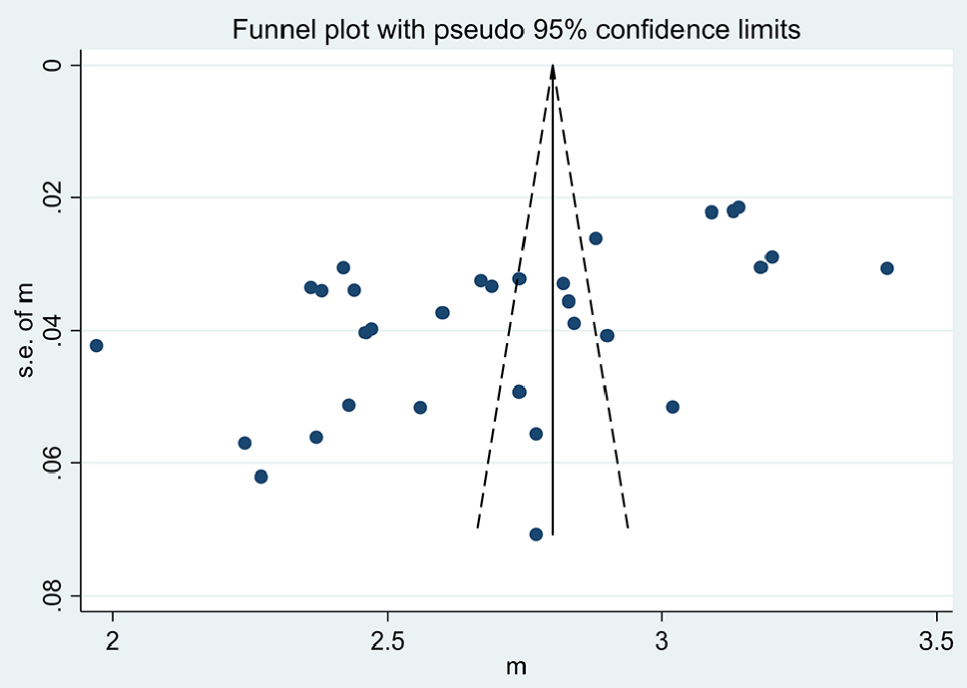
The publication bias of the average score of organizational silence evaluated through a funnel plot

To assess publication bias, we performed funnel plot, Begg’s test and Egger’s test. The funnel plot revealed a roughly symmetrical distribution of the included studies on both sides, indicating a low possibility of publication bias in this outcome. However, we observed a few scattered points at the bottom of the inverted funnel plot (see Fig. 4), suggesting a potential influence of small sample size on the outcome. Egger’s test result was 0.001 and Egger’s test result was 0.016. Therefore, the results of publication bias indicated that this study may have been influenced by publication bias.

## Discussion

This is the first quantitative meta-analysis of organizational silence among clinical nurses involving 34 moderate or high-quality cross-sectional studies, including 13,394 participants from 3 countries. The results of the meta-analysis revealed a moderate level of organizational silence among nurses with the score of 2.69 (95% *CI*: 2.57–2.81), which indicated that appropriate measurements should be took to stop the situation from getting worse.

### Current status of nursing organizational silence

The meta-analysis results from this study indicate that nursing organizational silence is at a moderate level overall. Consistent with Yao Xin’s [29] study but higher than Seren’s [30] study results. This may be attributed to differences in healthcare environments since Seren’s study primarily focused on public hospitals in Turkey. In addition, Sarıboğa et al. [31] found low levels of organizational silence among 330 teachers in İstanbul, which is lower than those of nursing organizations. This could be explained by the fact that nursing is usually linked with high intensity and stress, and in such a work setting, nurses may choose silence to minimize additional stress and conflict [32].

Furthermore, subgroup analysis revealed that clinical nurses in China and Korea had relatively high levels of organizational silence, which may be attributed to the interplay of traditional cultural norms and nursing practice systems. First, the ethical values of traditional Confucianism impact nurses’ acquiescent silencing practices via the conflict avoidance mechanism. Confucianism, for example, emphasizes the ideals of “harmony is precious” and “silence is golden,” as well as the repression of individual expression in order to maintain collective harmony [33]. This cultural tendency is replicated in nursing practice through opinion retention in hierarchical communication [34]. An qualitative study of 15 nurses from various provinces in China discovered that nurses’ quiet was one of the most common coping strategies when confronted with conflict [35].

In terms of different departments, the study revealed that pediatric nurses had the lowest scores in organizational silence. It’s possible that the pediatric work environment is more easygoing and dynamic, encouraging nurses to share their ideas. In addition, study [36] discovered that pediatric nurses have relatively high job satisfaction, are more satisfied with the work environment and management style, and believe they can be respected and recognized, so they are more willing to express their ideas and opinions rather than remain silent. This shows that there is a need to foster open communication in the workplace in order to facilitate discussion and exchange among nurses [24].

### Sources of heterogeneity

Since high heterogeneity was found in this analysis (*I*^*2*^ = 98.9%, *p*<0.001), we performed subgroup analysis, meta-regression, and sensitivity analysis to explore the reason for the heterogeneity.

Subgroup analysis was carried out, and we found that the oncology department was able to explain the source of heterogeneity partially. This could be due to the unique nature of oncology care, which involves multidisciplinary collaboration and necessitates frequent interactions between nurses, doctors, and pharmacists [37, 38], and this flat communication structure may weaken hierarchical authority and reduce defensive silence. Furthermore, the high psychological stress and complex situations of oncology patients led nurses to be more likely to commence vocalization in order to optimize nursing judgments [39]. Therefore, it is particularly important to establish a multichannel communication system to guarantee that nurses have prompt and correct access to organizational information as well as the chance to successfully convey their ideas and perspectives to leaders and colleagues [40]. In addition, strategies should be developed to transform nurses’ psychological stress into a positive voice [41].

Meta-regression analysis with year of publication, sample size, and assessment scale as covariates revealed that none of the variables mentioned above could be used to explain the source of heterogeneity (*P* > 0.05). Sensitivity analysis had also not yet revealed a source of heterogeneity. However, this does not rule out the possibility that those factors are unrelated to the current state of organizational silence among nurses, indicating that more high-quality studies should be conducted in the future to analyze the influence factors of organizational silence among nurses. Moreover, factors such as work environment [16, 42], nurse leaders’ management style [43, 44], personality characteristics among nurses [45], years of experience [46], and occupational behavior [17, 47] of the nurses should be considered in the investigation about the level of organizational silence.

In conclusion, the high heterogeneity observed in this study could be attributed to significant heterogeneity in the status quo meta-analysis, as well as the fact that cross-sectional studies are constrained by study design, sample selection, implementation, and evaluation to avoid measurement and selection bias. Future research should quantify unmeasured confounding variables using prospective cohort studies or mixed-methods approaches.

### Impact and recommendations for intervention

A wide number of studies have discovered that organizational silence can have a negative impact on individual nurses, healthcare organizations, and patients [48, 49]. However, a small number of studies have revealed that moderate organizational silence can help to maintain excellent interpersonal connections, minimize poor communication and resource waste, and have other positive effects in specific cultural situations [20, 21, 50]. For example, in the operating room, where nurses must work closely together, moderate organizational silence might prevent team conflicts caused by over-expression of personal opinions, thereby boosting team collaboration. However, excessive organizational quiet can lead to information not being shared in a timely manner and organizational problems not being resolved [11, 32]. Therefore, it is critical to assess the degree of organizational silence among clinical nursing nurses at the appropriate time [6]. Nursing managers should select relevant and effective assessment tools and develop tiered management plans for different degrees of organizational silence to maximize the positive benefits of organizational silence.

### Limitations and future research

This study has several potential limitations. First, Egger’s and Begg’s tests indicate a potential publication bias, which could be attributable to small-sample study effects, selective publication processes, or other factors in organizational silence research. Despite the inclusion of random-effects models and sensitivity analysis to limit the influence of bias, the present evidence may still overestimate the combined effect of organizational silence in nurses, therefore its practical significance should be evaluated with caution. Second, the included studies were cross-sectional in design, so causality could not be inferred from observed associations, and inevitably suffered from design flaws. Lastly, studies that just took into account research written in Chinese and English might have missed research written in other languages.

Our investigation has unveiled three potential avenues for future research. Firstly, forthcoming studies should use intervention research or mixed methods to delve into responsive strategies that address organizational silence. Secondly, as culture emerges as a crucial factor influencing nurses’ expression or silence, it is imperative to conduct cross-cultural research on organizational silence to explore the underlying reasons for nurses’ silence and the impact of diverse cultural backgrounds on organizational silence. Besides, it is also crucial to recognize the potential positive effects of nurses’ organizational silence. Previous studies [51] have demonstrated that moderate silence can be beneficial. Therefore, exploring how to harness the positive aspects of nurses’ organizational silence becomes a worthwhile topic to investigate.

## Conclusion

In conclusion, this review found moderate levels of organizational silence among clinical nurses. Subgroup analysis revealed that oncology as a cause of heterogeneity and pediatric nurses had the lowest levels of organizational silence in comparison, indicating that there is still space for improvement in organizational silence among nursing organizations. In light of this, nursing management should be fully aware of the potential impact of organizational silence on individual nurses, nursing organizations and the quality of patient care. Therefore, there is a need to develop and implement targeted management strategies that balance the encouragement of vocalisation with situational tolerance of silence to improve nurses’ agency and the quality of patient care.

## Electronic supplementary material

Below is the link to the electronic supplementary material.

Supplementary Material 1

## Data Availability

The author confirms that all data generated or analyzed during this study are included in this published article.
