# Supplementary material for: Heeding the voices of nurses: a systematic review and meta-analysis of organizational silence levels among clinical nurses
Source: BMC Nurs. 2025 May 16;24:552. doi: 10.1186/s12912-025-03138-1 (PMC12084937; doi:10.1186/s12912-025-03138-1)
Supplement: Supplementary file 1 — Supplementary Material 1 [file 12912_2025_3138_MOESM1_ESM.docx]

**Supplementary Materials**

Appendix 1: Search strategies of each database.

Appendix 1: Search strategies of each databas

|  | |
| --- | --- |
| **Database** | **Index and keyword terms** |
| PubMed | ((((((Nurses[Title/Abstract]) OR (nurse[Title/Abstract])) OR (Nursing Personnel[Title/Abstract])) OR (Personnel, Nursing[Title/Abstract])) OR (Registered Nurses[Title/Abstract])) OR ("Nurses"[Mesh])) AND ((organizational silence) OR (organizational silent)) |
| Web of science | TOPIC: (nurses OR nurse OR Nursing Personnel OR Personnel, Nursing OR Registered Nurses) AND TOPIC: (organizational silence OR organizational silence) |
| EMBASE | ('nurses':ab,ti OR 'Nursing Personnel':ab,ti OR 'Personnel, Nursing':ab,ti OR 'Registered Nurses':ab,ti) AND ('organizational silence':ab,ti OR 'organizational silenct':ab,ti) |
| CINAHL | (TI nurses OR TI nurse OR TI Nursing Personnel OR TI Personnel, Nursing OR TI Registered Nurses) AND (TX organizational silence OR organizational silent) |
| Cochrane Library | #1 MeSH descriptor: [Nursing] explode all trees  #2 (nurses or nurse or Nursing Personnel or Personnel, Nursing or Registered Nurses):ti,ab,kw) |
|  | #3：#1 or #2  #4 (organizational silence or organizational silent):ti.ab,kw  #3 and #4 |
| CNKI | (TI = ('组织沉默') OR KY = ('组织沉默') OR TKA = ('组织沉默') OR FT=('组织沉默')) AND (TI = ('护士'+'注册护士'+'护理人才') OR KY = ('护士'+'注册护士'+'护理人才') OR TKA = ('护士'+'注册护士'+'护理人才') OR SU = ('护士'+'注册护士'+'护理人才')) |
| WANFANG | (主题:(护士)) and (主题: (组织沉默) |
| VIP | (M=(组织沉默) OR R=(组织沉默) OR T=(组织沉默) OR U=(组织沉默)) AND (M=(护士 OR 注册护士 OR 护理人才) OR R=(护士 OR 注册护士 OR 护理人才) OR T=(护士 OR 注册护士 OR 护理人才)) |
| CBM | (("注册护士"[常用字段:智能] OR "注册护士"[全部字段:智能] OR "注册护士"[标题:智能] OR "注册护士"[摘要:智能]) OR ("护士"[常用字段:智能] OR "护士"[全部字段:智能] OR "护士"[标题:智能] OR "护士"[摘要:智能] OR "护理人才"[常用字段:智能] OR "护理人才"[全部字段:智能] OR "护理人才"[标题:智能] OR "护理人才"[摘要:智能])) AND ("组织沉默"[常用字段:智能] OR "组织沉默"[全部字段:智能] OR "组织沉默"[标题:智能] OR "组织沉默"[摘要:智能]) |
|  | |
